# An Investigation into the Prevalence of Methamphetamine Related Enquiries to Local Government Environmental Health Officers

**DOI:** 10.3390/ijerph21040455

**Published:** 2024-04-08

**Authors:** Emma J. Kuhn, Kirstin E. Ross, G. Stewart Walker, Jackie Wright, Harriet Whiley

**Affiliations:** 1 College of Science and Engineering, Flinders University, Adelaide, SA 5001, Australia; kirstin.ross@flinders.edu.au (K.E.R.); stewart.walker@flinders.edu.au (G.S.W.); jackie.wright@flinders.edu.au (J.W.); harriet.whiley@flinders.edu.au (H.W.); 2 Environmental Risk Sciences, Sydney, NSW 2118, Australia

**Keywords:** methamphetamine contamination, environmental health officer, local government, testing, remediation, thirdhand exposure, regulation, guidelines, public health

## Abstract

Methamphetamine contamination of residential properties remains a serious public health concern for members of the public. External stakeholders including Environmental Health Officers (EHOs) and testing and remediation technicians are engaged on investigating whether contamination has occurred from manufacturing or smoking processes. More specifically, local council EHOs are responsible for managing clandestine drug laboratories when notified by police and also for responding to public enquiries. However, the full scope of these contaminated properties is not seen by any single stakeholder, making it very challenging to quantify these situations. To evaluate the prevalence of methamphetamine related enquiries from the general public to EHOs, this study surveyed and interviewed officers from around Australia. It was found that public enquiries were infrequent with only 6% of respondents having received enquiries in the last month, which indicates that people are seeking information from other sources. Interestingly, there were case study scenarios that also mentioned issues with awareness and the flow of information. Concerns regarding difficult cases, police notifications, and site visits were also highlighted. The results of this study provide a benchmark of how methamphetamine related cases are managed and highlight the need for trustworthy information that is available to EHOs, governments, industry members, and the public in a unified location.

## 1. Introduction

Illegal drug production is a significant issue worldwide [1]. In particular, it has been shown that the methamphetamine market is continuously increasing [1]. Concerningly, it is estimated that only 1 in 10 clandestine drug laboratories are discovered by law enforcement [2]. Methamphetamine is an amphetamine-type stimulant that is found in several different forms: a waxy substance, a white powder, and a crystalline form [3,4]. Of the extensive list of illicit drugs that can be manufactured, methamphetamine is of particular relevance to public health due to the potential long-term contamination arising through its manufacturing and smoking processes [5,6]. Methamphetamine contamination of residential properties can occur in cars, holiday homes, workplaces, and even public spaces [7,8,9,10]. Consistent exposure to methamphetamine can lead to Thirdhand Exposure to Methamphetamine (THEM) syndrome, which is a compilation of health effects including respiratory issues; itchy, watery eyes; skin irritation; and behavioural issues [5].

Environmental Health Officers/Practitioners (EHOs/EHPs, referred to as EHOs from here on) are the front-line educators and regulators of public health for local government [11]. They manage an array of issues in local government areas including food safety, immunisations, mosquito management, septic tanks, swimming pools, and building contamination associated with clandestine drug labs [12,13]. Health protection departments from around the world recognise the challenges associated with clandestine labs and methamphetamine contamination [14,15,16,17,18,19,20]. While many of the recommendations are issued from individual states within nations, it can also be difficult to ascertain who accepts responsibility for cases involving methamphetamine contamination. Currently, in Australia, when a council is notified of a clandestine drug lab by police, an EHO will manage the potential health issues associated with property contamination by using regulatory tools such as Public Health Acts or Residential Tenancies Acts [21]. However, if a resident suspects methamphetamine contamination in their home from a previous homeowner, tenant or visitor, this contamination is not captured under similar regulatory tools. Thus, the management of these cases can vary depending on the state, territory, and council location. This study investigated EHO responses to methamphetamine contamination of homes and analysed some of the challenges that were raised by local government EHOs in Australia. This information will inform the development of future guidelines and professional practice in managing the increasing risk of THEM.

## 2. Materials and Methods

A mixed-method approach that followed an explanatory sequential design was used to collect information about current EHO professional practice related to methamphetamine contamination. This included an online survey with quantitative and qualitative questions, followed by phone interviews that were qualitative in nature. This study was approved by the Flinders University Human Research Ethics Committee (HREC) (Project number 5574).

### 2.1. Survey Participants

Two methods of participant recruitment were used for the survey. The first was to invite attendees of the Environmental Health Australia (EHA), the national Environmental Health professional body, at the national conference held in Tasmania in September 2022 to participate. To ensure that the survey was also available to those that had not attended the EHA conference, an email was sent out after the conference to all EHA members, inviting them to participate. The survey link remained open for 21 days to ensure that participants could complete the questions without time pressure.

### 2.2. Survey Questions

The survey was a short, targeted survey that consisted of nine questions in a combination of multiple choice, order of prevalence, and free-text opportunities for extended answers. Qualtrics^®^ (Provo, UT, USA) is an online platform that was utilised to create and host the survey data. A Quick Response (QR) code was generated for participants to access the survey on their mobile devices. The questions are available in the Supplementary Data S1.

### 2.3. Phone Interviews

Participants of the online survey were asked whether they were willing to participate in a follow-up phone interview to share additional information on their experiences with regulating methamphetamine contamination in properties. The follow-up phone interviews were approximately 30 min in duration and followed eight scripted questions (S2). These interviews were initially transcribed by speech to text software Otter.ai^®^ (version 3.46.1-7898), and then imported into NVivo 12^®^, which is a qualitative data analysis software.

## 3. Results

### 3.1. Survey Participants

A total of 79 EHOs participated in the online survey, with representation from every state and territory across Australia with the exception of the Northern Territory. The highest number of responses came from Queensland (27%), followed by Tasmania (21%), South Australia (18%), New South Wales (14%), Western Australia (9%), Victoria (9%), and the Australian Capital Territory (2%).

### 3.2. Phone Interviews

Of the 79 survey respondents, 20 people that agreed to a phone interview in the survey and were subsequently contacted via email, of which 18 participants shared their experiences. Western Australia (7) had the highest number of interviewees, then Queensland (4), then Victoria (2), South Australia (2), Tasmania (2), and New South Wales (1). The interviews were allocated 30 min; however, some respondents were happy to discuss their experiences for longer. The following results present a combination of the survey and interview responses.

### 3.3. Public Enquiries

Separate to police notifications, only 6% of respondents stated that they had received 1–5 public enquiries about methamphetamine contamination in a month, and the remainder said that they do not receive them on a regular basis. It is likely that concerned residents are seeking information and advice elsewhere. When members of the public do make enquires, the most prevalent methods are email, website submission, phone, and then in-person.

EHOs were asked whether enquiries had increased during the COVID-19 pandemic. Eighteen percent said yes, which could be attributed to residents becoming more aware of suspicious activities in their neighbourhood due to the increased time spent at home during lockdowns [22,23]. There were also factors such as cost and public stigma that disincentivise people from reporting clandestine labs or suspected contamination. One EHO reported that due to the size of the council area, they had recruited a call centre that was given scripting to screen their phone calls.

“*They would more than likely be told (from the call centre staff) that unless they have an identified source, we would not take the complaint*.”

(Participant 1)

Another EHO stated

“*If we have a resident that is concerned that their neighbour is making meth or some weird odours, we get them to contact police first*.”

(Participant 3)

Similarly, other EHOs reported that they have had interactions with building inspectors that have used presumptive testing kits while surveying the structural integrity of a property.

“*I’ve had those sort of reports sent to me where they’re positive. And that is really tricky, because, you know, we know that this sort of limitations in terms of that sort of testing. And, you know, when it’s just that sort of testing that is been provided, it’s hard for us to do anything about that*.”

(Participant 2)

### 3.4. Council Notices

When a house has been reported as contaminated by police, EHOs should put a notice on the council’s property file that states the property must be tested and remediated. This is to ensure that the council members are aware that the property must be decontaminated prior to being sold or rented. The results show that 22% of interview participants stated that they were monitoring a vacant property that required remediation within their council area.

EHOs can initiate another process that attaches a notification to the property title which informs future owners of the previous works. This will also advise them if there is any outstanding work that is required. During an interview, an EHO in New South Wales highlighted that there are two different ways to attach it to a property title. There is a compulsory title, and a voluntary title. It is dependent on the lawyer or conveyancer which property title search they request, or whether they complete both.

“*If they only get the 1492 which is the compulsory one, they can miss out on the 1495 notification of the clan lab*.”

(Participant 5)

When a notation is added to a property file, it is not permanent. They can be removed from the file with no previous history. This issue was also raised by other EHOs in Victoria and Western Australia. If a record of these notices was kept, it could offer grounds for subsequent tenants to seek testing or remediation advice. Other EHOs mentioned property managers not checking the property file before having building contractors commence work, and potential buyers having to conduct multiple council searches to establish if their newly purchased property had been flagged as a clandestine lab.

### 3.5. Site Visits

There was also a divide between councils that chose to do site visits for clandestine labs and those that did not. Of the 10 councils answering this question, 80% said that they did not allow their officers to visit the property, while 20% said that they do go onsite. Councils that did go on site to clandestine labs would go to obtain photos of the drug paraphernalia as it was being removed by law enforcement, and to talk with the officers to gain any further information that could be used to support their public health notification on the property.

“*It really helps when you start getting all the reports coming from the consultants as well, it helps you be able to get your head around it and make sure that the clean-up and assessments have been done properly*.”

(Participant 5)

The main determining factor for attendance to onsite inspections was a concern for the officers’ safety. Many officers were a part of small teams or even sole EHOs for regional councils, thus having a secondary officer present was unfeasible.

“*Our attitude here is that we do not do onsite visits for clan lab jobs. It’s much safer that way*.”

(Participant 1)

### 3.6. Police Notifications

The survey results found that due to the inconsistent nature of clandestine lab notifications, some local council areas receive more than others. This results in a variation in clandestine lab case management experience. When EHOs were asked how many clandestine drug labs their council has handled in the last month, 14% selected 1–5, and 86% said that they do not receive them on a regular basis. These were total clandestine drug lab notifications from the police.

The different approaches to visiting the site or not were sometimes impacted by the lack of detail provided by law enforcement in their notifications. From the phone interviews, there was a divide between councils that maintained a good relationship with local law enforcement and those that did not. This was linked with receiving sufficient details in written clandestine laboratory notifications. Unfortunately for councils that do not have a connection with the local police officers, they are not permitted to call law enforcement for clarification if there are inadequate details in the notification.

“*My main issue is the fact that the notification process gives us so little information for myself and for any of my staff who are looking to attend (the site)*.”

(Participant 4)

### 3.7. Difficult Cases

Survey participants were asked if they had ever experienced a difficult case or one that could not be resolved. There were 58% that said yes, and 42% said no.

Many of these cases involved the homeowner facing criminal charges and being in jail. In this instance, it is difficult to obtain information on who will be managing the contaminated property.

“*The simplicity of having the ability to get in contact with that person or have a law enforcement member find out and get information of who is going to deal with it on the criminal’s behalf*.”

(Participant 4)

Of the phone interviews, 27% said that they were monitoring vacant properties from cases that were not resolved. They would drive past and sometimes discuss the vacancy with the surrounding neighbours to ensure that no people moved in while the house was still contaminated.

“*One (clandestine lab) was in a caravan that was located inside a shed at the back of a residential property. So we touched base with the Department of Transport with our concerns. If they just drive away with this van, we’ll never know where the van is ever again. So we called them. This was all complete news to them, they basically had no real responsible knowledge of what can or should happen*.”

(Participant 6)

This participant described a scenario involving a mobile clandestine lab resulting in a contaminated car. They reported that in this instance, the Department of Transport and the insurance company were both highly proactive and had the vehicle written off and destroyed. However, this scenario raised an emerging issue of contaminated cars and caravans, which are currently not captured under any of the currently guidelines of regulations.

## 4. Discussion

It has been established that EHOs are often under-recognised for the multifaceted work they perform to protect public health (Whiley et al., 2018). This could be contributing to the lack of awareness from the general public on who they should contact if they suspect methamphetamine contamination of their property, whether it be from a clandestine lab or due to smoking. The results of this study suggest that members of the general public are not fully aware of the role of local government EHOs in this space. This is a concerning issue that needs to be addressed as it may be further exacerbating the problem of under reporting. This would result in many cases not being investigated and, therefore, not appearing in data or statistics. It would be highly beneficial to have a national or state campaign to raise awareness in communities on the role of EHOs, especially with respect to methamphetamine contamination.

### 4.1. Issues with Consistency

The main finding of this study was that there is little consistency in the management of clandestine labs and methamphetamine contamination cases, both within and between states and territories. This issue can be partially attributed to the bimodal distribution of council experience related to methamphetamine contamination of properties, meaning that they were either highly experienced or had very little to no experience. This poses a challenge for the profession and local governments, as it can be difficult to maintain the knowledge skill set if it is not consistently utilised, or if the experienced or knowledgeable EHOs leave the organisation.

The absence of clear and detailed directives is another contributing factor. Currently in Australia, there are the National Clandestine Drug Lab Remediation guidelines and the Voluntary Code of Practice for the assessment, remediation, and validation of former clandestine drug laboratories and other methamphetamine contaminated properties [21,24]. The national guideline provides foundational information that is applied Australia-wide; however, it is focused on labs detected by law enforcement and has broad recommendations that can be open to interpretation. The voluntary code of practice provides guidelines on the finer details involved in assessment and remediation for members of the testing and remediation industry. Enquiries about methamphetamine contamination, however, require a slightly different scope which has not been captured or focused on in the same way.

### 4.2. Governmental Responsibilities

enHealth are the national body that develops guidance for Australian health protection departments related to environmental risk factors that impact human health. Health protection departments are within the state government network and maintain strong connections with local government. The main responsibility for local government is to decide how to regulate according to what is suitable for their community and council members [25,26]. How they choose to engage with their constituents and how they manage any enquiries varies with each local government. This is leading to inconsistencies in the profession, with many potential financial and public health consequences. As such, there is a need to generate a standard approach that encompasses all aspects of methamphetamine contamination to ensure consistent messaging is given to all regulations, industry members as well as the general public, and other external stakeholders.

The enHealth guidelines for *Legionella* control provide a good example of how a national approach has been established and used in conjunction with state and territory based guidelines [27]. For example, South Australia have the Guidelines for the Control of *Legionella*, which are focused on the installation, maintenance, testing, decontamination, and inspection of manufactured water systems [28]. Western Australia has taken the approach of developing the Prevention and control of Legionnaires’ disease Code of Practice. This document covers the same key sections as the South Australian guidelines, but also includes potting mix and soils, and occupational safety and health regulations [29]. Thus, each state and territory have their own regulations and legislation to support their EHOs to manage this environmental health risk, but they are based on a nationally consistent enHealth framework.

The process and management of a clandestine lab or contaminated property has a similar structure, with the national framework and then guidelines coming from the states and territories. The Australian Clandestine Drug Laboratory Remediation Guidelines provide some guidance, and the enHealth document considers the public health perspective. Yet, this study shows that there needs to be improvement in the level of detail that is comprehensible to members of the public that are concerned not just about clandestine labs but also about contamination from smoking [21,30]. The main issue is that these guidelines are broad in nature and too generalised to be utilised. More specific information and guidance is required to ensure that they are beneficial and translatable to those that seek to use it.

### 4.3. Collaboration

Open communication between councils and law enforcement is essential for a good working relationship. Kacperska and Łukasiewicz [31] have described knowledge sharing between individuals as being integral for any organisation, and as being invariably linked with trust and efficiency. Similar to hoarding and squalor cases, which are also managed by local government EHOs, clandestine labs are multifaceted situations which often include financial problems, child and/or animal neglect, and public health safety concerns [32,33,34]. For hoarding and squalor cases, many of these problems arise from a combination of physical impairment and mental health issues [35]. Contact with these services is initiated by EHOs; however, many stakeholders are required to be involved to resolve a hoarding and squalor case; these include (but are not limited to) rubbish removal contractors, law enforcement, and community health services [9,36]. As with hoarding and squalor cases, when dealing with methamphetamine contamination, there needs to be a more consistent approach. This should be applied to the information that is provided by police, to engagement with other services, and also to ensuring that these services understand the role that EHOs have.

## 5. Conclusions

The purpose of this study was to understand the prevalence at which members of the public contacted the EHO at their local council. The results indicate that the general public are seeking their information from sources other than local government, and they also highlight the variability in case management. It can be concluded that while some good processes are in place, a more streamlined approach is ideal for EHOs, members of the public, and those in the decontamination industry.

## 6. Recommendations

This study highlights that a national framework for EHOs and heath protection departments would assist in streamlining the management of methamphetamine contaminated properties. This would need to be all encompassing and should include contamination arising through smoking, previous contamination of unknown origin identified by the general public, and contamination of mobile assets such as vehicles and caravans.

In particular, the participants of this study stated that it would be highly beneficial to have an approved list of testers, remediators, and occupational hygienists. Currently, Western Australia is the only state that provides a publicly available list of approved contractors for methamphetamine decontamination. This is an interesting approach given that there is no regulation or accreditation for this industry. Some states have legislation that prevents them from recommending companies for any services, which ensures that there is no bias or conflicts of interest. A regulatory body, adequate training, and an accreditation process are necessary to act as a benchmark for those working within industry. This will ensure a fair course of action is taken to help support members of the public and others seeking an experienced and qualified professional. Consistent information and transparency will build trust for the remediation industry members, within in the profession, and in the community.

To address some of these concerns, there is a need for a toolkit that can be used by EHOs, law enforcement, and the general public to provide consistent information and a clear process to follow. It is evident that some members of the public are unsure of who to contact; therefore, website information that outlines the differences between clandestine labs and smoking can assist those in need. Ideally, an accessible platform should be populated with helpful information and maintained to ensure that the content is readily available and remains current. This proposal could begin with a single state and then expand to both align the current practices and also meet the needs of other states and territories to ensure consistency.

## 7. Limitations

This study provides insight into local government EHO interactions with members of the public about methamphetamine contamination, but it does not represent all council areas. In conjunction, the number of responses varied between states, thus further research in this area should be conducted to provide a more robust and unified answer to some of these questions.

## Data Availability

Data is not publicly available, however it can be provided by contacting the corresponding author.

## Supplementary Materials

The following supporting information can be downloaded at: https://www.mdpi.com/article/10.3390/ijerph21040455/s1, Data S1: Survey questions; Data S2: Interview questions.
