# Supplementary material for: An Investigation into the Prevalence of Methamphetamine Related Enquiries to Local Government Environmental Health Officers
Source: Int J Environ Res Public Health. 2024 Apr 8;21(4):455. doi: 10.3390/ijerph21040455 (PMC11050614; doi:10.3390/ijerph21040455)
Supplement: Supplementary file 1 [file ijerph-21-00455-s001.zip › EHO paper Supp/Data S1 EHO survey.pdf]

EHO Survey

Start of Block: Default Question Block

Q1 This research project has been approved by the Flinders University Human Research Ethics Committee (Project 5574). For queries regarding the ethics approval of this project, please contact the Executive Officer of the Committee via telephone on
+61 8 8201 2543 or email human.researchethics@flinders.edu.au

Q2 Participation for this survey is voluntary. You may choose not to answer any questions and are free to withdraw at any time.

Q15 Please read this Letter of Introduction for this research

Q4 Please read this Information Sheet for further details about this online survey.

End of Block: Default Question Block

Start of Block: Block 1

Q5 This survey is based on your current council area.

Q6 Your state

- ◯ Western Australia (1)
- ◯ Northern Territory (2)
- ◯ South Australia (3)
- ◯ Queensland (4)
- ◯ New South Wales (5)
- ◯ Australian Capital Territory (6)
- ◯ Victoria (7)
- ◯ Tasmania (8)

Q7 2. How many queries about methamphetamine contamination do you receive per month?

- ◯ I do not receive them on a regular basis (1)
- ◯ 1-5 (2)
- ◯ 5-10 (3)
- ◯ 10-20 (4)
- ◯ 20+ (5)

Q8 3. How have you received these enquiries? Select in order of prevalence (1-5)
______ Email (1)
______ Website submission (2)
______ Phone (3)
______ In person (4)
______ Other (5)

Q9 Have you had cases that are difficult or cannot be resolved? If so, please describe.

____

____

____

____

____

Q10 5. Do you think that Covid-19 has increased the frequency of methamphetamine contamination enquiries?

- ○ No (1)
- ○ Yes - reason for your answer (2) ____

Q11 6. How many former clandestine laboratories (seized by police) has this council handled in the last month?

- ○ I do not receive them on a regular basis (1)
- ○ 1-5 (2)
- ○ 5-10 (3)
- ○ 10-20 (4)
- ○ 20+ (5)

End of Block: Block 1

Start of Block: Block 2

Q12 7. Would you like to participate in a phone interview to provide more depth to your answers?

○ No (1)

○ Yes (2)

Q13 8. If you selected yes for a phone interview, could you please provide contact details and I will be in contact shortly.

○ First name (1) ____

○ Phone number (2) ____

○ Email (3) ____

○ Preferred days and time to be contacted (4) ____

Q14 9. Would you like to be informed of the outcome of these survey results? If yes, please state if you would prefer a short summary and/or a copy of the publication.

○ No (1)

○ Yes (2) ____

End of Block: Block 2
