# Supplementary material for: An Investigation into the Prevalence of Methamphetamine Related Enquiries to Local Government Environmental Health Officers
Source: Int J Environ Res Public Health. 2024 Apr 8;21(4):455. doi: 10.3390/ijerph21040455 (PMC11050614; doi:10.3390/ijerph21040455)
Supplement: Supplementary file 1 [file ijerph-21-00455-s001.zip › EHO paper Supp/Data S2 EHO interview.pdf]

Methamphetamine contamination EHO Interview

This interview is based on your current council area.

1. Do enquiries usually disclose why they believe there is methamphetamine contamination?

   Yes

   Sometimes

   No

2. When speaking to public, what are most common questions about?

   Clandestine labs (manufacture)

   Personal use (smoking)

   Businesses that provide testing services

   Businesses that provide remediation services

   Other

3. Does your council recommend any businesses to the public that offer testing or remediation services?

   Yes

   No

4. Have you handled a complaint or had negative experiences with businesses that provide testing and/or remediation services?

   Yes

   No

5. How often has this occurred?

   I do not receive them on a regular basis

   1-5

   5-10

   10-20

   20+

6. Do you have a particular case that you found challenging that you would like to share with me? Please describe.

7. Do you feel like you have the tools to assist members of the public with methamphetamine contamination related enquiries?

   Yes
   No – please explain

8. Do you have any suggested improvements or comments about how methamphetamine contamination is handled on a council level?

   No
   Yes – please explain
